# Contributions of Adaptive Laboratory Evolution towards the Enhancement of the Biotechnological Potential of Non-Conventional Yeast Species

**DOI:** 10.3390/jof9020186

**Published:** 2023-01-31

**Authors:** Ticiana Fernandes, Carolina Osório, Maria João Sousa, Ricardo Franco-Duarte

**Affiliations:** 1 CBMA (Centre of Molecular and Environmental Biology), Department of Biology, University of Minho, 4710-057 Braga, Portugal; 2 Institute of Science and Innovation for Bio-Sustainability (IB-S), University of Minho, 4710-057 Braga, Portugal

**Keywords:** non-*Saccharomyces*, non-conventional yeast species, adaptation, evolution, biotechnology, yeasts

## Abstract

Changes in biological properties over several generations, induced by controlling short-term evolutionary processes in the laboratory through selective pressure, and whole-genome re-sequencing, help determine the genetic basis of microorganism’s adaptive laboratory evolution (ALE). Due to the versatility of this technique and the imminent urgency for alternatives to petroleum-based strategies, ALE has been actively conducted for several yeasts, primarily using the conventional species *Saccharomyces cerevisiae*, but also non-conventional yeasts. As a hot topic at the moment since genetically modified organisms are a debatable subject and a global consensus on their employment has not yet been attained, a panoply of new studies employing ALE approaches have emerged and many different applications have been exploited in this context. In the present review, we gathered, for the first time, relevant studies showing the ALE of non-conventional yeast species towards their biotechnological improvement, cataloging them according to the aim of the study, and comparing them considering the species used, the outcome of the experiment, and the employed methodology. This review sheds light on the applicability of ALE as a powerful tool to enhance species features and improve their performance in biotechnology, with emphasis on the non-conventional yeast species, as an alternative or in combination with genome editing approaches.

## 1. Introduction

One of the main problems regarding the future sustainable use of the Earth’s resources is the extensive use of petroleum in modern-day life. The current concerns and debates about economy and environmental protection encourage a transition towards eco-friendly and eco-efficient societies, detached from economic growth, through the increased use of finite global resources to green chemical production processes. This would promote economically viable and safe industrialization that will be of foremost importance for a new worldwide equilibrium. Harboring vast phenotypic and genetic diversity, microorganisms can make an important contribution to the development of greener processes, fostering innovation and helping in this transition to more sustainable industries. As complex entities, microorganisms have been carefully selected according to multiple criteria and used as key players in the most diverse fields from food and beverage production to the development of pharmaceuticals and different added-value chemical products, among others. Furthermore, numerous attempts have been made to improve their performance, aiming at different applications, with Adaptive Laboratory Evolution (ALE) being one of the most explored in recent years.

The concept of adaptive evolution (also called natural evolution) dates back to Darwin and has been clearly understood and noticed in nature, consisting of the appearance of random mutations and recombination in organisms’ genomes, followed by natural selection that will favor the most adapted (the fittest) organisms in specific environmental conditions and in a certain timeframe. When performed in the context of a specific population and under the controlled conditions of a scientific experience, this process is named Adaptive Laboratory Evolution (ALE). Several reviews are available covering general aspects of ALE [1,2,3,4] or focused particularly on the obtention of specific evolved traits [5,6,7]. However, the efficiency and applicability of ALE towards the industrial evolution of non-*Saccharomyces* yeasts have not yet been discussed, and so the current review focuses on the particular difficulties of evolving non-conventional yeasts and on the advantages of using these evolved yeasts in industrial biotechnological applications. Additionally, we summarized and discussed new studies that illustrate ALE as fruitful for the enhancement of yeast traits considering both phenotypic and genotypic dynamics and compared this method with new approaches employing genome editing and sequencing.

## 2. Yeasts as Attractive Biological Models for Adaptive Laboratorial Evolution

The fast progress in fields such as genetics, biology, and engineering has increased the interest in yeasts with enhanced traits to be further employed in the most diverse research, industrial, and commercialization areas. In many contexts, the term “yeast” has been traditionally used to refer to *Saccharomyces cerevisiae*, as this species has been employed worldwide for alcoholic fermentation and baking almost exclusively. However, +1500 yeast species belonging to +150 genera have been described [8]. Recently, Drumonde-Neves et al. [9] reviewed 80 years of literature about wine yeasts, concluding that 293 different yeast species are associated with winemaking, in addition to the traditional *S. cerevisiae*. For example, *Torulaspora delbrueckii* stands out today as one of the most interesting so-called non-*Saccharomyces* yeast species, bringing a wide array of beneficial flavor and aromatic characteristics to wine [10,11]. The ease with which genes can be manipulated also makes yeast diversity attractive as a biological resource to transfer specific metabolic pathways to *S. cerevisiae* and establish reliable models to predict phenotype–genotype interactions [12,13]. Currently, some genetically modified wine yeasts are commercially available [14,15,16]. However, the market value of these strains relies on each country’s current legislation regarding the use of genetically modified organisms (GMOs) in food products, together with a lack of consumer acceptance. It has already been discussed by several authors that the universal acceptance of these genetically altered organisms so that they can achieve a significant place in the market will take a long time if it ever occurs [17,18,19]. In this way, a search for alternatives has been pursued by several researchers worldwide. With the potential to overcome this dilemma, ALE has emerged as a successful tool to study evolutionary phenomena occurring in microorganisms in controlled laboratory conditions, helping to gain insight into basic molecular mechanisms and, simultaneously, to obtain strains with improved phenotypes [3]. This approach involves the inoculation of microorganisms under specific selective conditions for long-term adaptation, over many generations—lasting for weeks, months, or even years—through a continuous increase in selective pressure (for example, the concentration of a specific compound or via the use of stressful growth conditions). The fitness of the population is shaped by the competition for limited resources and the capacity to successfully transmit the beneficial mutations to the following generations [3]. During microorganisms’ adaptation to the surrounding environment, strains can undergo several mutations that can be categorized as beneficial, neutral, deleterious, or lethal. Since experiments are designed to direct the selection in a particular way, deleterious mutations are eliminated, and population evolution is driven by beneficial mutations. These mutations, observed while microorganisms evolve during many generations, can be analyzed in terms of single nucleotide polymorphisms (SNPs), smaller insertions and deletions (InDels), and larger deletions and insertions, which will then be connected to gene regulatory and fitness modifications during the selection for improved phenotypes [3]. William Dallinger [20] is considered the first scientist to perform ALE experiments through the exploitation of the influence of slow increases in temperature (from 15.56 °C up to 70 °C) on the survival of monads, whose life cycles were relatively short, in a seven-year study from 1880 to 1886. With this thermal study, Dallinger showed that all evolved organisms were able to survive at 70 °C, in opposition to the initial ones. Reports of ALE employing *S. cerevisiae* and *Escherichia coli*, the most well-known species within the yeast and bacteria groups, respectively, alongside microalgae, viruses, and mammalian cell lines, have grown exponentially in recent years [3,21,22,23,24,25,26,27].

## 3. Current Status and Applications of ALE in Biotechnology

The main advantages of ALE involve the ease of implementation of the method, the fact that it does not require any prior knowledge on genotype–phenotype interconnections, and the capacity to exploit phenotypes that require the combination of several intracellular pathways, such as stress tolerance and rapid cell growth, with this being a powerful adjunct to metabolic engineering [28]. On the other hand, there are some inherent limitations associated with this practice such as the extensive time consumption of the methodology, the need for continuous and laborious monitorization of the cells, the requirement for a direct connection between the desired feature and a benefit to the microorganism, and the need for an extreme asepsis environment to avoid culture contaminations. The optimization of metabolic pathways, the improvement of fermentation rates, the emergence of resistant and tolerant phenotypes, the development of new organoleptic features, aroma innovations, greater uptake rates, morphology modifications, environmental adaptations, and the improvement of lipids accumulation are some of the features that are presently being exploited in the scope of this evolution-based strategy. It should be noted that often the selection of evolved variants, in a particular environment, leads to significant trade-offs in alternative conditions. In this line, the choice of the best phenotype for a particular biotechnological process has to weigh strains’ performances, but also the least trade-offs associated. An important detail in the search for ALE-enhanced phenotypes is the time span for the selection experiment, with a typical range varying somewhere between 100 and 2000 generations. Moreover, it is possible to obtain a fitness increase of up to 50–100% between the 100th and 500th generations, which corresponds to approximately 2 months of selection for a typical culture of *S. cerevisiae* or *E. coli*, then decreasing considerably during the course of ALE [3].

### 3.1. Experimental Approaches of ALE

**Figure 1:**
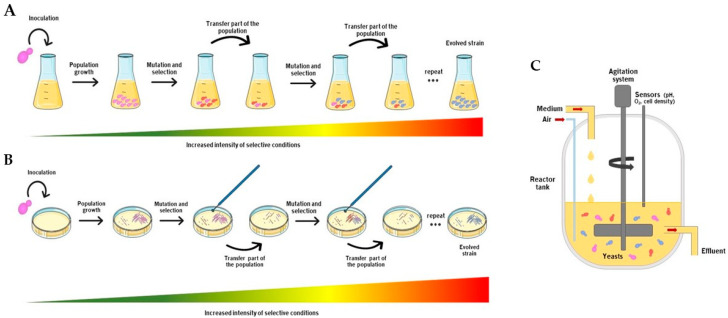
Schematic overview of the experimental serial batch culture approach. (**A**) Microorganisms are cultured in flasks with liquid media, chosen to mimic a desired selective condition, for an extended period of time, and aliquots of the cells’ population are sequentially transferred to fresh media. Cells will undergo recombination and mutations, over time, generating evolved strains. (**B**) Colony transfer is a similar process to liquid transfer but is performed in solid medium plates. (**C**) Continuous cultures are usually carried out in chemostat tanks, in which fresh medium is introduced at a stable flow rate and effluent fluid emerges at the same rate, maintaining constant environmental culture conditions.

A proper experimental design is the foundation of a successful ALE experiment. Several mechanisms and strategies were tested to evolve microorganisms in the laboratory, from serial to continuous experiments (Figure 1). General considerations of these approaches are discussed in the sections below and summarized in Table 1.

#### 3.1.1. Serial Batch Cultures

Serial batch cultivation is the simplest and most popular ALE procedure. It is usually employed when the selective environment does not require constant conditions, as evolutionary constraints such as culture pH and nutrient composition will change during the culture growth. The implementation of this experiment involves the serial transfer of an aliquot of the population into new media containing the selective pressure condition [29]. Furthermore, it could be performed in two different batches: Liquid or solid media. In a liquid environment (Figure 1A), cells are inoculated in flasks with fresh medium to promote their growth with subsequent cultivation under the desired selective pressure. Afterward, a fraction of the culture is transferred into a fresh medium with a slight increase in the selective pressure until the appearance of a desirable mutant that will expand within the rest of the population over time. The total number of generations, although a concept that is often hard to quantify in this type of experiment, allows one to estimate the emergence of adaptive mutations, and cell densities within 10^7^ and 10^9^ cells per mL are commonly used. Choe et al. [22] suggest the following equation to calculate the number of cell divisions:

$$\mathrm{Number}\;\mathrm{of}\;\mathrm{generations}=\log_{2}\frac{\mathrm{Final}\;\mathrm{cell}\;\mathrm{density}}{\mathrm{Initial}\;\mathrm{cell}\;\mathrm{density}}\;$$

Regular monitoring of cell viability, cell morphology, and residual media composition should be employed during ALE experiments to not only verify if a desirable mutant has emerged but also to check for possible contaminations [29].

**Table 1:** Main applications, advantages, and disadvantages associated with different ALE approaches: Serial batch, continuous culture, and automated methods.

	Main Applications	Advantages	Disadvantages	References
**Serial Batch**	- Used when the selective environment does not require constant factors	- Simplicity to set up and use; - Use of low-cost equipment; - Requires only one operator; - Possibility to parallelize	- More susceptible to random drift; - Difficulties to continuously and frequently monitor cells; - Higher susceptibility to errors; - Harder to execute (periodic transfers); - Maintenance of the exponential growth and appropriate selective pressure level	[3,29,30,31]
**Continuous culture**	- Used to maintain the constant physiological state of microorganisms; - Used in mutation accumulation studies	- Large population sizes; - Less drastic reduction of the population size; - Control of the evolving population-specific growth rate; - Variety of systems	- High cost of the operation	[3,29,31,32,33,34]
**Automated methods**	- Applied in batch and continuous cultures; - Complex culturing procedures	- Allow cell transfers 3 to 7 times more frequent than the manual method and at a constant growth phase; - Reduce the possible fluctuations in the size of the transferred volume; - Variety of systems	- High cost of the equipment; - Computationally demand	[28,29,31]

During the evolutionary process, aliquots of the evolving populations are usually stocked in glycerol (within 10% to 40%, *v/v*) and maintained at −80 °C for further phenotypic analysis [35,36,37]. The primary advantages are that this strategy is very easy to set up and use, requires low-cost equipment, can usually be executed by a single operator, and can be adapted to massive parallel cultures [3,4,5,6,7,8,9,10,11,12,13,14,15,16,17,18,19,20,21,22,23,24,25,26,27,28,29,30], as summarized in Table 1. However, batch cultures are more susceptible to random drift due to certain shortfalls, which include variations in the population density, growth rates, and different nutrient supplementations, together with fluctuations in environmental conditions [29].

In solid media, ALE is performed using colony transfer and plating (Figure 1B), where yeasts grow on agar plates and one or more (successfully grown) colonies are randomly chosen and periodically transferred to a new plate with fresh medium, for several generations. However, the use of such a small population hampers the selection process by increasing the chance of genetic drift in determining allele frequencies (reviewed in Swamy and Zhou [33]), with this method being used less, in comparison with evolution in liquid medium.

Batch cultivation during long periods of time imposes some problems that usually increase the chances of failure: (i) Difficulties in continuously and frequently monitoring cells; (ii) higher susceptibility to errors, due to the periodic transfers of cells and media; (iii) complications in the maintenance of the exponential growth; (iv) difficulties in keeping the appropriate selective pressure level. However, these complications can be managed through the recent introduction of full or partial automation, as reviewed by Sandberg et al. [31] and further discussed in Section 3.2.

#### 3.1.2. Continuous Culture

The use of continuous systems, or chemostats, is the second most popular culturing technique for ALE experiments [38]. This approach is recommended when a constant physiological state is intended, i.e., to maintain a stable population density and growth rate via a constant influx of media at a set dilution rate [3,29,31,32]. Continuous systems are composed of bioreactors, which enable tight control over environmental parameters such as pH and oxygenation. A major drawback is the expensive cost of operation as it requires a substantial investment in the initial equipment. On the other hand, this system benefits from the increased tolerance to larger population sizes, smaller bottleneck effects, and the possibility to control the specific growth rate. The growth kinetics of the culture depends on the concentration of the limiting substrate and of any growth inhibitors [29].

The chemostat strategy is widely applied for mutation accumulation studies (Swamy and Zhou [33]), and additional variations are available such as retentostat—a chemostat with a filter in order to retain the biomass on the efflux—and auxostat—a chemostat with an additional feedback mechanism that controls cell density. Furthermore, the latter can be subdivided into at least four variants: Turbidostat, pH-stat, oxistat, and morbidostat. The turbidostat concept leans on the control of the cell density through variations in the dilution rate, while the pH-stat is distinguished by being based on deviations in the pH of the culture. The last two methods—oxistat and morbidostat—endorse the management of the culture’s conditions via dissolved oxygen and pH, density, or optical density, respectively [34]. Additionally, Ekkers et al. [34] suggested a bioreactor system entitled omnistat, which is highly flexible and involves lower costs, and can be used in many bioreactor configurations. This system can be pre-programmed to both temporal and spatial variations to accurately induce a selective regime suitable for the specific research question. The omnistat can be configured to implement various alternative bioreactor modes, such as the ones mentioned above.

### 3.2. Automated Methods for ALE: High-Throughput Adaptive Evolution

Recently, there has been a growing demand to automate adaptive evolution processes, both using batch and continuous cultivation, in order to surpass difficulties allied with these methodologies. The manipulation by a machine allows the transfer of batch cultures to be carried out with a frequency of 3 to 7 times higher than the manual procedure, allowing the transfer of the cells in a constant growth phase, reducing possible fluctuations in the size of the transferred volume and preventing the loss of beneficial mutations [28]. One such technique is entitled eVOLVER, a scalable and automated culture system that allows the precise and multiparameter regulation of growth conditions. eVOLVER was the first automated system available to perform ALE and has the huge advantage of being cost-effective and quickly re-configured to virtually implement any type of high-throughput growth experiment [39]. This system enables ongoing control and monitoring of hundreds of individual cultures by collecting, measuring, and recording experimental data in real-time, for any time scale, allowing one to aim for desired parameters such as temperature, culture density, and media composition. The performance of this method can be surpassed through its conjunction with a genetic system designed for targeted mutagenesis of user-selected genes in vivo (OrthoRep—orthogonal DNA replication), giving rise to the automated continuous evolution (ACE; [40]). This platform specifically targets the evolution of biomolecules. In this technique, the OrthoRep works as an orthogonal DNA polymerase-plasmid that mutates the genes of interest approximately 100,000-fold faster than the average mutation rates typically associated with the host genome in vivo, while eVOLVER’s robust framework ensures experimental durability over long timeframes. The combination of these two approaches assures the speed, depth, and scale of the continuous evolution of the ACE strategy [40]. Due to the complexity of ALE studies, and to optimize and design the experiments, a computational framework was developed: ALEsim [41]. With this strategy, resources can be deployed in an optimized way at different steps of the experiment in order to reduce the project timeline and accomplish the desired outputs. Perceiving the distance to optimality can help researchers to determine when to cease an ALE experiment. The most frequent mechanism of determining when to terminate an ALE trial is to assess that no more improvements in fitness are being detected.

Uncovering the genotypic basis of a particular feature is one of the main goals of exploiting adaptive evolution, and the loss of advantageous mutants imposes a restriction on discernible adaptive mechanisms. Winkler et al. [29] developed the pioneer system VERT, in which one can attempt to visualize evolution in real time, with applicability to isolate the evolved mutants. This method enables the visualization of competition between genotypes during evolution, relying on the use of distinct fluorescent strains to visualize expansions of fluorescently market subpopulations. Changes in the proportions of distinct subpopulations, so-called adaptive events, illustrate the emergence and expansion of evolved mutants with higher fitness advantages, in comparison with the background. The strain that will be used (parental strain) for the adaptive experiment has to be marked with different fluorescent proteins, which can be generated by using classic clonal methods. VERT provides a set of measurements that reflect the relative abundance of cells expressing different fluorophores (e.g., green fluorescent protein—GFP, yellow fluorescent protein—YFP, and red fluorescent protein—RFP) within the evolving population. The authors also developed an algorithm to computationally identify frequency modifications corresponding to adaptive events.

Table 1 summarizes the discussed experimental ALE approaches, comparing the advantages and disadvantages associated with each of the aforementioned methods.

## 4. Evolution of Non-Conventional Yeast Species through ALE

In contrast to traditional comparative genomic studies, ALE allows us to clearly associate phenotypic alterations with changes in the cultivation conditions, which, in turn, can be correlated with genomic alterations using whole-genome sequencing. Microorganisms are well-suited for evolution studies as they offer easy manipulation, controllable culture conditions, short generation time alongside large population sizes, and simple nutrient requirements [3,28]. Bacteria and yeasts play a central role in these studies and have been extensively exploited over the years. Bacteria have the advantage to be easily grown to high yields, displaying a vast library of studies available, comprising a wide choice of cloning strategies, and their gene expression can be efficiently controlled. However, despite the promising exploitation studies reported in the literature in recent years, bacteria are very sensitive to low pH and the presence of sulphur dioxide in the media, do not display post-translational modifications, have a small size and a low density, and are associated with a larger set of pathogenic strains [42]. In contrast to prokaryotes, yeast-based strategies are economically superior because yeasts are highly tolerant to lower pH environments, are larger in size, allow post-transcriptional modifications of proteins (i.e., glycosylation), have increasing genetic mutant libraries and omics repertoires, and have effective adapting abilities to stress conditions and are generally recognized as safe (GRAS) [43,44,45,46,47,48,49], reinforcing their potential as an asset model for the most diverse fields. Through ALE strategies, *S. cerevisiae* strains were successfully evolved, considering a panoply of biotechnological important traits: (i) Huang and Kao [50] and Randez-Gil et al. [51] successfully evolved *S. cerevisiae* strains into higher thermotolerant strains that were able to grow and develop at 40 and 42 °C, respectively; (ii) Cadière et al. [52] increased the carbon flux through the pentose phosphate pathway, increasing fermentation rates and different volatile aromas; (iii) Pereira et al. [53] exploited tolerance mechanisms with heightened acid tolerance by the *S. cerevisiae* strains understudy; (iv) De Vero et al. [54] obtained a reduction of sulphites (<10 mg^−1^) and H_2_S production levels; (v) Novo et al. [55] enhanced CO_2_ production, obtaining inferior sugar amounts at the end of fermentation, while also allowing faster fermentation kinetics, etc. Due to the versatility of ALE, studies have also been actively conducted for several non-conventional yeast species. The studies evolving non-conventional yeast species cataloged according to the aim of the study but also specifying the yeast species used, the outcome of the experiment, and the method employed are detailed in Table 2. In particular, the great majority of the studies compiled aimed to increase resistance to a certain compound and/or condition (ethanol, oxidative stress, and drugs) or increase the production of an added-value product (lipids, succinic acid, glycerol). In fact, microbial ALE applications in the biotechnological field can generally be classified regarding the following goals: (1) The activation of latent metabolic pathways; (2) increasing the tolerance to a particular substrate; (3) enhancement of strains’ fitness; (4) evolution of microorganisms to solve environmental constraints; and (5) phenotype optimization. Additionally, Slininger et al. [56] describe other goals of ALE, beyond those presented in this review.

The development of industrial strains intends to maximize productivity (product formation in unit time) and yield (product formation per substrate consumption) of the process [28]. The activation of a latent pathway is important due to the growing number of chemicals that are currently produced by biological systems from renewable resources. This not only reduces our dependency on fossil fuels but also moves us towards a fully circular economy and bio-sustainable society [28]. Phenotype optimization through ALE has demonstrated that the adaptation of growth-coupled metabolic engineering designs can lead to a significant increase in the production rate and a reduction in by-product formation [78,79,80]. Moreover, the adaptation to a minimal growth environment, usually performed by gradually reducing the number of supplements from passage to passage while cells remain in the exponential phase, can significantly simplify downstream processing, increase substrate and product tolerance, and reduce the cost of large-scale production [80].

Regarding yeast species, Table 2 shows that *Yarrowia lipolytica* and *Candida* spp. seem to be the preferred species for evolving experiments, even though a large diversity of species and genera have been used in recent years. Once considered spoilage organisms, many non-*Saccharomyces* species, the so-called non-conventional yeasts, have recently been pointed out as auspicious agents for effectively contributing with improved traits and promoting competitiveness in several processes, including the fermentation of several beverages and the production of bread and other diverse food products [9,10]. The downside of some non-*Saccharomyces* yeast species is the release of high levels of volatile acidity, low tolerance to high ethanol content, and the reduced knowledge available in comparison to the standard yeast, *S. cerevisiae*. The implementation of unconventional yeasts has been outlined in several locations worldwide, as a great number of these species introduce novel sensory profiles of beverages, contribute a complex aromatic matrix to the final product such as in wine and beer, decrease the spoilage risk, and complement *S. cerevisiae* performance [9].

One of the most desirable traits attempted to be improved by ALE has been increased ethanol resistance by non-conventional yeasts. From the several strategies applied primarily to *T. delbrueckii* [36], *Kluyveromyces marxianus* [59,60], and *Barnettozyma californica* [61], the most successful ones reported an increase in resistance in 11.5% (*v/v*) ethanol in evolved strains of *T. delbrueckii* [36], and 122% higher specific growth rates of *K. marxianus* strains in 4% (*v/v*) ethanol [60]. One interesting aspect of ALE is the occurrence of multiple evolved phenotypes, as an indirect result of a particular directed evolution towards a certain goal. For example, Phommachan et al. [66], aimed to use strains of *Candida tropicalis* to improve their resistance to high glucose concentrations. After long-term cultivation with increasing temperatures (40 °C to 44.5 °C) and the periodical transfer of evolved cultures to media with high glucose concentrations, authors obtained strains that, in addition to fermenting high glucose concentrations, also showed increased tolerance to ethanol, furfural, and hydroxymethylfurfural at high temperatures, and improved xylose-fermenting ability. However, ALE is also exploited as a proof-of-concept strategy. It has been proposed that the use of sulphite-based preservative agents—commonly used to inhibit the growth of spoilage microorganisms—may have contributed to the selection of better-adapted *Brettanomyces bruxellensis* strains to survive in winemaking environments. To support this proposal, Bartel et al. [68] utilized ALE to show a clear evolution of isolates when exposed to sublethal contents of sulphites, also suggesting that the mechanism responsible involves the gene *SSU1*. Guo et al. [81] analyzed two *Dekkera* species—*D. bruxellensis* and *D. anomala*—and identified 12 vacuolar H ^+^ -ATPase (V-ATPase) genes that were under positive selection, which aid in developing tolerance to high alcohol and high sugar pressure. Additionally, the authors demonstrated that the PGK1 enzyme is responsible for the increased glycolysis rate. These outcomes suggest an efficient translation system developed by *Dekkera* yeasts to promote adaptive evolution.

## 5. ALE Versus Genome Editing

Great advances have been made to expedite the ALE workflow, namely in combination with genome editing strategies in order to exploit the phenotypic–genotypic relations leading to species improvement. Genetic engineering involves the construction of organisms to produce targeted products by either introducing exogenous genes or knocking out native genes, which requires prior knowledge of the organisms’ genome. However, ALE is a practice that allows the redirection of metabolism without the need to know a priori the genome of the target mode. As practical consequences, strains evolved through ALE can be used to improve crop yields, reduce food waste and costs, and treat diseases, among others. The major drawback is associated with the long duration that ALE experiments take to actually obtain improved strains [82].

Lately, some studies have emerged taking advantage of the synergistic combination of ALE and metabolic engineering to use yeasts as cell factories to improve their features [83]. Narisetty et al. [83] expressed a heterologous acetyl-CoA synthase in a *Y. lipolytica* strain and later subjected the constructed strain to ALE to improve its tolerance to increasing contents of acetate as the sole carbon source. The authors not only successfully evolved this yeast to resist high concentrations of acetate but also detected an enhancement of succinic acid production (5.1–6.5 g/L) with this strategy. Baek et al. [84] developed D-Lactic-acid-producing *S. cerevisiae* strains through metabolic engineering by expressing the highly stereospecific D-lactate dehydrogenase gene from *Leuconostoc mesenteroides* subsp. *mesenteroides* ATCC 8293 in *S. cerevisiae* lacking natural lactic acid production activity. D-lactic acid production was increased by reducing the release of ethanol and glycerol. The engineered strain was then subjected to ALE in order to enhance LA tolerance alongside the overexpression of an HAA1 gene involved in the lactic acid stress response resulting in a production of up to 48.9 g/L. Lee et al. [85] integrated two copies of a mutant xylose isomerase into *S. cerevisiae* alongside gre3 and pho13 deletion, showing the overexpression of both native xylulokinase (XKS1) and heterologous transaldolase (tal1) from *S. stipitis*. The resultant engineered yeast displayed an improved xylose fermentation performance with a yield of 0.45 g ethanol/g xylose. Another example combining both strategies involved the coupling of a biosensor with an anti-metabolite selection scheme in *S. cerevisiae* to screen for improved muconic acid production [86]. Ito et al. [87] designed a random genome-disruption library to identify gene-disruption-type effective factors that enhance the secretory production of targeted proteins, with *Komagataella phaffii* used as the host strain. From here, the authors identified six factors for which disruption led to increased antibody production. Afterward, multiple gene knockouts were applied, complementing the study with ALE strategies to recover the reduced cell growth promoted by the gene knockout. Perli et al. [88] showed that the *Ogataea parapolymorpha* Moco biosynthesis pathway combined with the expression of a high-affinity molybdate transporter could lead to the synthesis of Moco in *Y. lipolytica*. Furthermore, ALE was required to increase the growth of yeasts on nitrate resulting in an increase of up to 100-fold of nitrate reductase activity and a 4-fold increase in the growth rate (reaching 0.13 h^−1^). Other authors have recently exploited the advantages of combining ALE and engineering strategies for different genera of microorganisms, particularly aiming to improve the production or consumption of different compounds [89,90,91,92,93,94]. 

In summary, despite the promising outcomes proven by metabolic engineering studies with significant increases in product titers, this methodology also introduces higher production costs and complexities to large-scale production and demands for specialized knowledge and expertise. On the other hand, ALE stands out as a superior strategy since it does not require prior knowledge of the genome of the organism under study, allows the recovery of fitness loss from engineering strategies that may introduce further unintended mutations, and, above all, is suitable to contour the low level of public acceptance of GM for food production by encouraging more “natural” ways to enhance yeasts performance [95,96].

## 6. What Is Next for ALE? The Growing Compromise between Evolution and Technology

The complete genome of an organism is generally considered a map of all the features encoded within the DNA. However, knowledge of the genome sequences does not directly outline how this genetic information induces observable behaviors. Comparative genome sequence analysis could be a major asset in ALE studies, particularly to discriminate conserved from divergent DNA segments, or even functional sequences from non-functional ones, between species that undergo changes under the same stressful environment. The breakthrough that technology has experienced recently has led to the emergence of new high-tech approaches in bioinformatics, genetic engineering, and improved microbiology culture methodologies, including next-generation DNA sequencing (NGS) and transcriptional and metabolomic profiling, which have allowed for the birth of a new science: Omics. These advances made it possible to outline novel phenotypes through the use of biological models. Natural production hosts are reviving researchers’ interest in increased productivity due to the boost in available yeast genome sequences, based on omics data analysis and metabolic modelling. Transcriptomics, proteomics, metabolomics, and genomics are branches of omics that support the underlying knowledge of phenotype–genotype correlations and help unlock downstream effects evidenced by the complex networks present in the cell. Although major sub-network and pathways are often well conserved among distinct yeast species, up- and downstream connections that separate cellular networks differ significantly between them, leading to species-specific variances [97]. As mentioned in previous sections, mutations are the underlying basis of genetic modifications and are distributed as SNPs—contributing 61% of the majority of observed phenomenon, 29% of deletions, 3% of insertions, and 3% of insertion sequence movements, as reviewed by Conrad et al. [98]. 

During an ALE experiment, there is direct competition between a parental strain and an adapted variant, in which the latter presents improved fitness that will be obvious as a result of its increased frequency within the total population [3,99]. Powerful algorithms have been developed to be able to elucidate beneficial mutations based on physiological modifications at a distinct status, making ALE more accessible for broad phenotypes with fewer human interventions, although in this case, it requires greater machine power that is not always accessible to all laboratories [82,100]. Despite the increasing availability of engineering tools, determining the causality of the appearance of a phenotype is still a time-consuming process. It is in this context that NGS plays an important role by easing the process of discovering mutations from evolved genomes, although the challenge remains to try to entail particular genotype components to phenotypes [101,102]. However, there is also a need to understand and overcome the bottlenecks of cellular and metabolic pathways. Multi-omics characterization together with systems modelling approaches allow the interpretation of molecular-level data and direct deciphering of motifs for the adaptation of evolved phenotypes or reconstructed microorganisms, based on alterations in the values of singular transcripts, proteins, or metabolites, revealing possible targets for future engineering of improved microorganisms. In short, the integration of omics data with genomic-scale models provides a context in which these data can be integrated and interpreted [82,103]. Additionally, computational techniques such as machine learning will be valuable in unveiling relationships between different phenotypes, contributing to the systematic knowledge of microbial metabolism and gene regulation [12,104].

## 7. Conclusions

Although considerable progress has been made in genetic engineering, the low acceptance of products obtained with this approach in the market makes ALE a suitable and current topic with the potential to be explored in an industrial context. Therefore, the possibility to evolve the great genetic and phenotypic diversity already present in non-*Saccharomyces* species enhancing attractive features without the need to manipulate organisms’ genomes would be a major asset to future applications.

## Figures and Tables

**Table 2:** Summary of evolution-based strategies employing non-*Saccharomyces* yeasts, including aim, outcome, and method employed in each study.

Aim of Study	Yeast Species	Outcome	Method	References
Develop the ability to use CO_2_ as carbon source	*Komagataella pastoris*	Growth rate increased from 0.008 to 0.018 h^−1^	Serial batch cultivations using minimal YNB medium for 27 to 29 generations	[57,58]
Enhance ethanol resistance	*Torulaspora delbrueckii*	Improved ethanol tolerance from 9% to 11.5% (*v/v*) and greater SO_2_ resistance	Serial batch cultivation in YPD medium supplemented with increasing ethanol contents (3, 6, 9, 10, 11, 12, and 14% (*v/v*)) for 114 days. Transfers of $1\times 10^{5}$ cells/mL when yeasts reached the mid-log phase	[36]
	*Kluyveromyces marxianus*	Ethanol tolerance increased up to 10% (*v/v*).	Cultures inoculated in increasingly ethanol concentrations (0, 4, 5, 6, 7, and 8% (*v/v*)) for 100 days, approximately 450 generations	[59]
		Strains with a 122% higher specific growth rate in 4% (*v/v*) ethanol	Four different populations cultured in 4 % (*v/v*) ethanol up to reach ~300 generations. Passage cultures were performed under ethanol stress for 85 days, a period in which there was a significant increase (above 50%) in the specific growth rate. Chemostat cultivation was also evaluated	[60]
	*Barnettozyma californica*	Improved ethanol production (4× higher than parental strain) and growth ability, enhancement of total sugar from 34 to 51.8 g/L and a twofold increase in nonvolatile toxic compounds such as phenol (1.017–2.11 g/L)	Sequential transfers with gradually increasing concentrations of 25, 50, 75, and 100% (*v/v*) of bagasse hydrolysate. Adaptation in each step was repeated 4 times	[61]
Enhance oxidative stress tolerance	*Candida glabrata*	Faster detoxification of H_2_O_2_ and increased growth ability	Three parallel populations of GFP and YFP-labeled cells were cultured in YNB medium and evolved in serial batch transfers using a periodic challenge strategy with H_2_O_2_ as a selective pressure for more than 180 generations	[50]
Increase tolerance to inhibitor compounds presented in hydrolyzed lignocellulosic substrates	*Rhodosporidium toruloides*	Increased growth, tolerance, and lipids production	Successive cultivations in increasing concentrations (increments of 10% (*v/v*)) of sugarcane bagasse hydrolysate in supplemental media	[62]
Acquisition of drug resistance to posaconazole	*Candida albicans*	Increases in drug tolerance to posaconazole, cross-tolerance to other azoles and widespread increases in genome size	Serial dilutions to fresh medium every 24 h for 4 days, at a ratio of 1:1000, for a total of ~50 generations of evolution. Performed for 12 replicate lines of each of the 8 strains understudy	[63]
Increase stress tolerance in response to lignocellulose-derived inhibitors	*Yarrowia lipolytica*	Strains with increased ferulic acid tolerance	Sequential transfers, every 48 h, of cells into a fresh medium with increased ferulic acid concentrations (0.5, 0.75, 1.0, and 1.5 g/L). Domestication lasted for 86 days, with approximately 57 generations	[64]
Acquisition of aneuploidy in the presence and absence of fluconazole	*Candida glabrata*	Exposure to fluconazole induced genome reorganization, some of which provided a fitness increase in the presence of the antifungal drug	A single colony was seeded in liquid culture for one round, overnight to grow, and then divided into 12 independent populations: half in the absence of fluconazole and the other half in the presence of the drug. Serial cultures were propagated for 330 generations of growth	[65]
Improve the sensitivity to high glucose concentrations	*Candida tropicalis*	Increased tolerance to ethanol, furfural, and hydroxymethylfurfural at high temperatures and improved xylose-fermenting ability and fermentation ability at high glucose concentrations	Long-term cultivation with increasing temperatures from 40 °C to 44.5 °C. Cultivation was performed two or three times at each temperature and lasted 7 days each. The culture which survived at 44.5 °C was transferred from low to high glucose concentration media several times	[66]
Comparative analysis of two ALE strategies (heterologous and colony, ALEh and ALEc, respectively) using hemicellulose hydrolysate as a selective pressure medium	*Rhodotorula toruloides*	Fitness gaining of 55%; lipid content of 64.3% in eucalyptus hemicellulose hydrolysate; higher biomass production (6.51 g/L) and a decrease of 4 h in lag phase	Sequential culturing in media with 5 consecutive increases in sugarcane hemicellulose hydrolysate (10% (*v/v*) per stage) and the addition of a colony selection step at the end of each stage	[67]
Sulphur dioxide resistance	*Brettanomyces bruxellensis*	Individual clones isolated from evolved populations exhibited enhanced sulphite tolerance (1.6 to 2.5 times higher than the corresponding parental strains)	Cultures that reached OD600 of 1.5–2.0 (∼7 generations) were subcultured (1 mL) into fresh media containing higher concentrations of sulphite. Sulphite content was increased by 0.03–0.06 mg/L mSO_2_ with every population subculture.	[68]
Biotethanol production	*Kluyveromyces marxianus*	Evolved strain showed a 3.3-fold higher specific growth rate; 56% reduced lag phase and 80% enhanced fermentation efficiency; ethanol titer and productivity obtained 54.8 g/L and 2.1 g/L/h, respectively.	Cultures were incubated at 42 ± 0.5 °C until log phase (OD_600_ of 0.6–0.8) with a gradual increase in inhibitor concentrations during repetitive batch cultures (acetic acid (A): 3.5–6; furfural (F): 2–3.2; vanillin (V): 2–3; cocktail (A + F + V): 3 + (0.3–1) + (0.3–0.8)).	[69]
Develop tolerance to ionic liquids (ILs)	*Yarrowia lipolytica*	Increased tolerance to high concentrations of ILs	Serial cultivation in 6-well plates with cells being transferred during mid-exponential phase into a fresh medium with sequential increasing concentrations of [EMIM] [OAc] to obtain a specific growth rate ≥ 0.02 1/h, during 200 generations	[70]
Increase in succinic acid production		Succinic acid productivity increased by 2.3-fold	Serial cultivation with increasing glucose concentration (25, 50, 75, 100, and 150 g/L). Population transfers were performed after cell growth achieved the exponential phase, lasting for 14 generations	[71]
Improve glycerol uptake rate and succinic acid release		Glycerol uptake rate increased by 13.5% and succinic acid productivity increased by 10%	Culture performed in a bioreactor with all conditions controlling maintained. When biomass reached 20 g/L, the culture medium was replaced by fresh medium (with an initial glycerol concentration of 100 g/L)	[72]
Multi-stress tolerance and increased ethanol production	*Kluyveromyces marxianus*	Adapted isolates gained the capacity for ethanol fermentation at high temperatures andimproved tolerance to multi-stress.	Sequential transfers with a gradual increase in temperature from 40 °C to 45 °C, at 160 rpm for 7 days. Cultivations were performed twice at each temperature.	[73]
Enhancement of ethanol production	*Spathaspora* *passalidarum*	Ethanol production (19.4 g/L) with productivity, yield, and xylose consumption rate of 0.8 g/L·h and 0.4 g/g, respectively, in a sugarcane bagasse hemicellulosic hydrolysate.	Serial batch cultivation with progressive increments of 10% (*v/v*) hydrolysate in each passage. Xylose concentration was maintained constant at 80 g/L for all combinations. Serial transfer of cell mass concentration to 5 g/L to the fresh medium was performed every 48 h until the medium was composed of hydrolysate only.	[74]
Optimize lipid production	*Metshnikowia pulcherrima*	Enhanced growth rates, reduced lag time, and increased lipid production	Cultures growth started in NLB + 0.6 g/L formic acid and nitrogen-limited broth inhibitor cocktail, with five replicate lineages for each condition. Culture transfers were performed after 48 h with the experiment ending after 1000 h	[75]
	*Yarrowia lipolytica*	30% higher lipid content	Two evolution experiments were performed in nitrogen and magnesium double-limiting medium, inoculating 3$\times 10^{8}$ cells. First: Growth at lipid storage until exhaustion and repeated for 3 rounds and 165 generations Second: Growth and lipid accumulation as the first set with the intermediate step of 2000 cells plated in carbon-free media with posterior transfer to PDB medium (repeated for 16 rounds and 105 generations)	[76]
	*Rhodosporidium toruloides*	Evolved strain displayed a 2.5-fold higher specific growth rate than the wild-type isolate.	Cultivation in YM broth supplemented with HMF (1.0 g/L) and furfural (1.0 g/L) during 16 sequential subcultures. Each subculture was initiated with OD = 0.1 and transferred when OD reached 15–20 within 48 h.	[77]
Ethanol production	*Scheffersomyces* *stipitis, Candida lusitaniae*	Improvement of ethanol production by *S. stipitis* and *C. lusitaniae* from 19.5 and 22.7 g/L to 21.4 and 23.9 g/L, respectively.	Cells were cultured in ten subcultures on YPHX during adaptive evolution, and yeast strains were incubated with agitation at 150 rpm for 24 h at 30 °C. Adapted cells were washed with fresh medium and transferred to water hyacinth hydrolysate medium for ethanol production.	[78]

## Data Availability

Not applicable.
